# A risk prediction model of perinatal blood transfusion for patients who underwent cesarean section: a case control study

**DOI:** 10.1186/s12884-022-04696-x

**Published:** 2022-04-30

**Authors:** Yao Wang, Juan Xiao, Fanzhen Hong

**Affiliations:** 1 Department of Obstetrics, The Second Hospital, Cheeloo College of Medicine, Shandong University, No. 247 Beiyuan Road, Jinan, 250033 Shandong China; 2 Center of Evidence-Based Medicine, The Second Hospital, Cheeloo College of Medicine, Shandong University, Jinan, Shandong China

**Keywords:** Perinatal blood transfusion, Postpartum hemorrhage, Cesarean section, Nomogram

## Abstract

**Background:**

Severe obstetric hemorrhage is a leading cause of severe maternal morbidity. A perinatal blood transfusion is the key factor in the treatment of severe obstetric hemorrhage. Our aim is to identify patients with a high risk of perinatal blood transfusions before Cesarean Section, which can promote the effectiveness of the treatment of severe obstetric hemorrhage, as well as improve obstetric preparations.

**Methods:**

This study retrospectively analyzed the data of 71 perinatal blood transfusion patients and 170 controls, who were both underwent Cesarean Section from July 2018 to September 2019. These data were included in the training set to build the risk prediction model of needing blood transfusion. Additionally, the data of 148 patients with the same protocol from October 2019 to May 2020 were included in the validation set for model validation. A multivariable logistic regression model was used. A risk prediction nomogram was formulated per the results of the multivariate analysis.

**Results:**

The strongest risk factors for perinatal blood transfusions included preeclampsia (OR = 6.876, 95% CI: 2.226–23.964), abnormal placentation (OR = 5.480, 95% CI: 2.478–12.591), maternal age (OR = 1.087, 95% CI: 1.016–1.166), predelivery hemoglobin (OR = 0.973, 95% CI: 0.948–0.998) and predelivery fibrinogen (OR = 0.479, 95% CI: 0.290–0.759). A risk prediction model of perinatal blood transfusions for cesarean sections was developed (AUC = 0.819; sensitivity: 0.735; specificity: 0.848; critical value: 0.287).

**Conclusions:**

The risk prediction model can identify the perinatal blood transfusions before Cesarean Section. With the nomogram, the model can be further quantified and visualized, and clinical decision-making can subsequently be further simplified and promoted.

**Supplementary Information:**

The online version contains supplementary material available at 10.1186/s12884-022-04696-x.

## Background

Severe obstetric hemorrhage is the leading cause of maternal death and severe maternal morbidity worldwide, especially in developing countries [1]. Severe obstetric hemorrhage mainly refers to severe postpartum hemorrhage (PPH), which is defined as persistent blood loss that is greater than 1500 mL within 24 h after delivery or the need for a blood transfusion for excessive postpartum bleeding [2]. Due to increased blood volume and enhanced coagulation functions during the third trimester, postpartum women develop a greater tolerance to blood loss; therefore, many symptoms of clinical blood loss can be easily masked, making it difficult to identify PPH at an early stage [3]. However, a perinatal blood transfusion is the key factor in the treatment of severe obstetric hemorrhage. Another main cause of perinatal blood transfusions is severe predelivery anemia, which can be improved by planned blood transfusions before Cesarean Section. The components of blood transfusions in obstetrics mainly include packed red blood cells (PRBCs), platelets, and fresh frozen plasma (FFP) or cryoprecipitate [4]. Due to the skewed estimation of blood loss and clinical symptoms in postpartum women, blood transfusions can be used to roughly assess the presence of the severe PPH.

The risk factors of severe PPH include uterine inertia, placental factors, soft obstetrical canal cracks, and coagulation disorders [5–7]. Undergoing a cesarean section is a predominant and independent risk factor for severe obstetric hemorrhage that has been well cited throughout the literature; thus, this procedure was the focus of this study [8–12]. It is important to identify a scientific method to predict the need for perinatal blood transfusions before cesarean section, which would ensure the improvement of obstetric preparations and optimization of resource allocation by health care institutes. Currently, there are no criteria for a prediction system for required perinatal blood transfusions. Although there have been several studies on the PPH risk prediction model, there is a lack of research on the role of perinatal blood transfusions within that model [13]. Furthermore, due to several differences in regions, races, economics, and social customs, among other factors, it is also necessary to develop an individualized risk prediction model to guide clinical treatment.

## Methods

This was a retrospective study that was performed in the Obstetrics Department of the Second Hospital of Shandong University, which is a Grade III general hospital in China in which the annual number of births exceeds 6,000 births every year. All the patient data that were extracted from the clinical electronic medical record system were deidentified, such that all private information was not included. All the procedures complied with the principles of the Declaration of Helsinki and were approved by the Ethics Committee of the Second Hospital of Shandong University. The requirement for informed consent was waived by the Ethics Committee because of the retrospective nature of the study.

### Definition of perinatal blood transfusion

In our study, the components of blood transfusion include PRBCs, platelets, and FFP or cryoprecipitate. The blood transfusion was defined as at least 2 units PRBC and/or 200 ml plasma and/or 1 unit platelet and/or 4 units cryoprecipitate transfusion. Besides, the time to prepare blood transfusion was intraoperative or postoperative within 24 h.

### Study population

Data were obtained from patients who had undergone cesarean sections in the Obstetrics Department of the Second Hospital of Shandong University from July 2018 to May 2020. The participants were eligible for inclusion if they were hospitalized due to cesarean sections at gestational age ≥ 28 weeks, at age ≥ 18 years, and did not meet any of the exclusion criteria. We excluded the following patients: those patients who underwent vaginal births after cesarean procedures; those patients who had missing information on blood transfusions; and those patients with missing information in their medical records.

After we applied the inclusion and exclusion criteria, 71 perinatal blood transfusion patients and 170 controls were recruited into the training set to build risk prediction model from July 2018 to September 2019. An additional file shows this in more detail [see Additional file 1]. Additionally, 49 blood transfusion patients and 99 controls were recruited into the validation set for the verification of the model from October 2019 to May 2020 using the same method [see Additional file 2]. There were no changes in protocols, blood products and patient population composition between the two periods. The decision to transfuse was at clinician discretion.

### Clinical characteristics and data sources

Clinical characteristics, which are based on prior known risk factors for PPH and clinical experiences in daily practice [7, 11, 14–16], can also be considered as candidate predictors of perinatal blood transfusions. The clinical characteristics included the following: maternal age at delivery and gestational age at delivery, whether live in local, whether have permanent job, number of previous deliveries, number of abortions, number of previous cesarean sections with anesthesia, gave birth during an emergency, whether predelivery oxytocin was used and pregnancy complications that included preeclampsia, hemolysis, elevated liver enzymes, and low platelets (HELLP) syndrome, gestational diabetes mellitus (GDM), macrosomia, placental abruption, abnormal placentation (including placenta previa and placenta increta), fibroid, polyembryony and coagulation disorders. These diseases were identified with the use of International Classification of Diseases 9^th^ *Revision, Clinical Modification* (ICD-9) diagnosis codes.

Data of the clinical characteristics were collected, and all the data were obtained from the electronic medical record system, which records the information of all hospitalized patients in the past 5 years in the Second Hospital of Shandong University, and these data can be verified by using hospital numbers.

### Laboratory examination

Predelivery hemoglobin, predelivery hematocrit, and predelivery platelet levels (the antenatal level that was most similar to the day of the cesarean section) were derived from routine blood results that were measured with the SYSMEX XN-9000 Automatic Blood and Body Fluid Analyzer (SYSMEX, Japan), Liver function tests were performed with a Roche 702 Biochemical Analyzer (Roche, Switzerland), Hepatitis B virus antigens and antibodies were measured with an Abbott Architect i2000 automatic immunoassay analyzer (Abbott, US).

### Statistical analysis

The continuous variables are presented as means and standard deviations or medians and range were compared by using Student's t tests or Wilcoxon signed rank tests. The categorical variables are expressed as absolute numbers with percentages, and Pearson’s chi-square tests or Fisher’s exact tests were used to compare the differences.

A multivariable logistic regression model was used to develop a prediction model for perinatal blood transfusions with the training set. A univariate analysis was first used; subsequently, the variables that were significant at a α level of 0.10 were included in the multivariate logistic model. A bidirectional stepwise elimination approach was used to simplify the model on basis of the Akaike information criterion. The variance inflation factor was used to measure multicollinearity, and a value > 10 was the criterion for the assessment of the multicollinearity of the factors.

A nomogram was formulated (per the results of the multivariate analysis) by using the rms package. To use the nomogram, the position of each variable on the corresponding axis was identified, a vertical line was drawn to the points axis for the number of points, the points from all the variables were summed, and a vertical line from the total points axis was drawn to determine the transfusion probabilities at the lower line of the nomogram [17].

The diagnostic value of the nomogram for the perinatal blood transfusions was expressed as the following values: sensitivity, specificity, critical value, and the area under the receiver operating characteristic curve (AUC). The bootstrap test (at 10,000 times) was applied to test the stability of the AUC. The comparisons of the AUCs were performed with the Z-score test.

All the statistical analyses were performed by using the rms and Statistical Product and Service Solutions (SPSS) packages. In the univariate analysis, the variables that were significant at a α level of 0.10 were included in the multivariate logistic model. The other statistical tests were 2-sided, and a *P* value < 0.05 was accepted as significant. Additionally, the confidence intervals for the proportions are reported as 2-sided exact binomial 95% CIs.

## Results

### Characteristics of the population in the training and validation sets

In the training set, 241 patients who underwent cesarean sections were included in this study: the average age was 31.61 ± 4.73 years, and the average gestational age was 272 days. When comparing the training set and the validation set, there were no significant differences in age, gestational age, the number of previous deliveries, the number of abortions, or the number of previous cesarean deliveries (Table 1).

**Table 1:** Characteristics of the study population in the training and validation sets

Variables	Training set (*n* = 241)	Validation set (*n* = 148)	*P*
Age, means ± SD (years)	31.61 ± 4.73	31.94 ± 4.92	0.509
Gestational age, Medians (lower quartile, upper quantile) (days)	272 (266–277)	271 (264–278)	0.610
Number of previous deliveries, n (%)			0.605
0	94 (39.00)	55 (37.16)	
1	130 (53.94)	80 (54.05)	
2	17 (7.05)	13 (8.78)	
Number of abortions, n (%)			0.863
0	109 (45.23)	71 (47.97)	
1	76 (31.53)	47 (31.76)	
2	38 (15.77)	20 (13.51)	
3 or more	18 (7.47)	10 (6.76)	
Number of previous cesarean deliveries, n (%)			0.931
0	117 (48.55)	71 (47.97)	
1	110 (45.64)	67 (45.27)	
2	14 (5.81)	10 (6.76)	

### Univariate analysis in risk predictors of the training set

In the univariate analysis, when the differences in blood transfusions and controls were compared, age, gestational age, anesthesia, predelivery hemoglobin levels, predelivery hematocrit levels, predelivery fibrinogen levels, preeclampsia, HELLP syndrome, placental abruption, abnormal placentation, polyembryony, and coagulation disorders were shown to be significantly associated with perinatal blood transfusions.

The average age of the transfused patients was 33.03 ± 5.56 years. The average gestational age was 36 weeks. Those who received transfusions were older, had lower gestational ages, and had higher proportions of general anesthesia, prenatal anemia, polyembryony, low predelivery fibrinogen levels, preeclampsia, HELLP syndrome, and abnormal placentation (Table 2).

**Table 2:** Predictors of perinatal blood transfusion in the training set

Variables	Transfusion (*n* = 71)	Control (*n* = 170)	t(Z)/Chi-square (Fisher)	*P*
Age, means ± SD (years)	33.03 ± 5.56	31.02 ± 4.23	2.729	0.007
Gestational age, Medians (lower quartile, upper quantile) (days)	252 (271–276)	273 (268–279)	-3.048	0.002
Anesthesia, n (%)				< 0.001
Spinal-epidural anesthesia	63 (88.7)	170 (100)		
General anesthesia	8 (11.3)	0 (0)		
Emergency	37 (52.1)	84 (49.4)	0.146	0.702
Predelivery hemoglobin, means ± SD (g/L)	109.89 ± 14.04	114.91 ± 12.17	-2.787	0.006
Predelivery hematocrit, means ± SD (%)	33.16 ± 4.08	35.34 ± 3.16	-4.109	< 0.001
Predelivery platelets, means ± SD (*10^9^/L)	205.85 ± 67.45	223.54 ± 54.05	-1.962	0.052
Predelivery fibrinogen, means ± SD (g/L)	3.87 ± 0.83	4.29 ± 0.68	-4.143	< 0.001
Preeclampsia, n (%)	14 (19.7)	6 (3.5)	17.248	< 0.001
HELLP, n (%)	4 (5.6)	0 (0)		0.007
GDM, n (%)	12 (16.9)	34 (20.0)	0.311	0.577
Fibroid, n (%)	6 (8.5)	7 (4.1)		0.212
Abnormal placentation, n (%)	26 (36.6)	13 (7.6)	30.995	< 0.001
Polyembryony, n (%)	6 (8.5)	0 (0)		< 0.001
Coagulation disorders, n (%)	3 (4.2)	0 (0)		0.025
Predelivery oxytocin, n (%)	7 (9.9)	10 (5.9)	1.208	0.272
Hepatitis B virus carrier or abnormal liver function, n (%)	1 (1.4)	5 (2.9)		0.673

Abnormal placentation includes placenta previa and placenta increta

### Multivariate logistic regression analysis in the training set

**Fig. 1:**
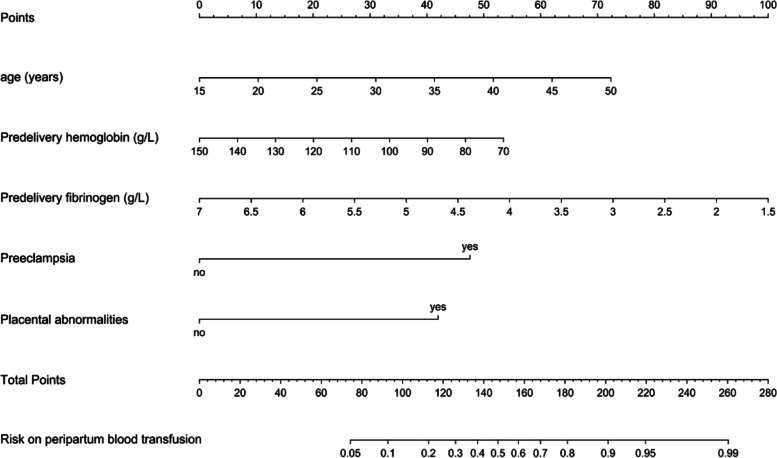
The nomogram for peripartum blood transfusion

In the multivariate analysis, the factors that were statistically significant in the univariate analysis were entered into the model. The model consisted of 5 risk factors: age, predelivery hemoglobin levels, predelivery fibrinogen levels, preeclampsia, and abnormal placentation (Table 3). These factors were applied in the construction of the nomogram (Fig. 1). In the bootstrapping validation, the nomogram demonstrated similar accuracy in predicting perinatal blood transfusions, with an AUC of 0.819. By using the receiver operating characteristic (ROC) curve, the best critical value of the “risk of perinatal blood transfusion” was 0.287.

**Table 3:** Multivariate model of peripartum blood transfusion in the training set

Variables	β	Odds ratio (95% CI)	*P* value
Age	0.084	1.087 (1.016, 1.166)	0.017
Predelivery hemoglobin	-0.027	0.973 (0.948, 0.998)	0.038
Predelivery fibrinogen	-0.737	0.479 (0.290, 0.759)	0.003
Preeclampsia	1.928	6.876 (2.226, 23.964)	0.001
Placental abnormalities	1.701	5.480 (2.478, 12.591)	< 0.001

### Model assessment

**Fig. 2:**
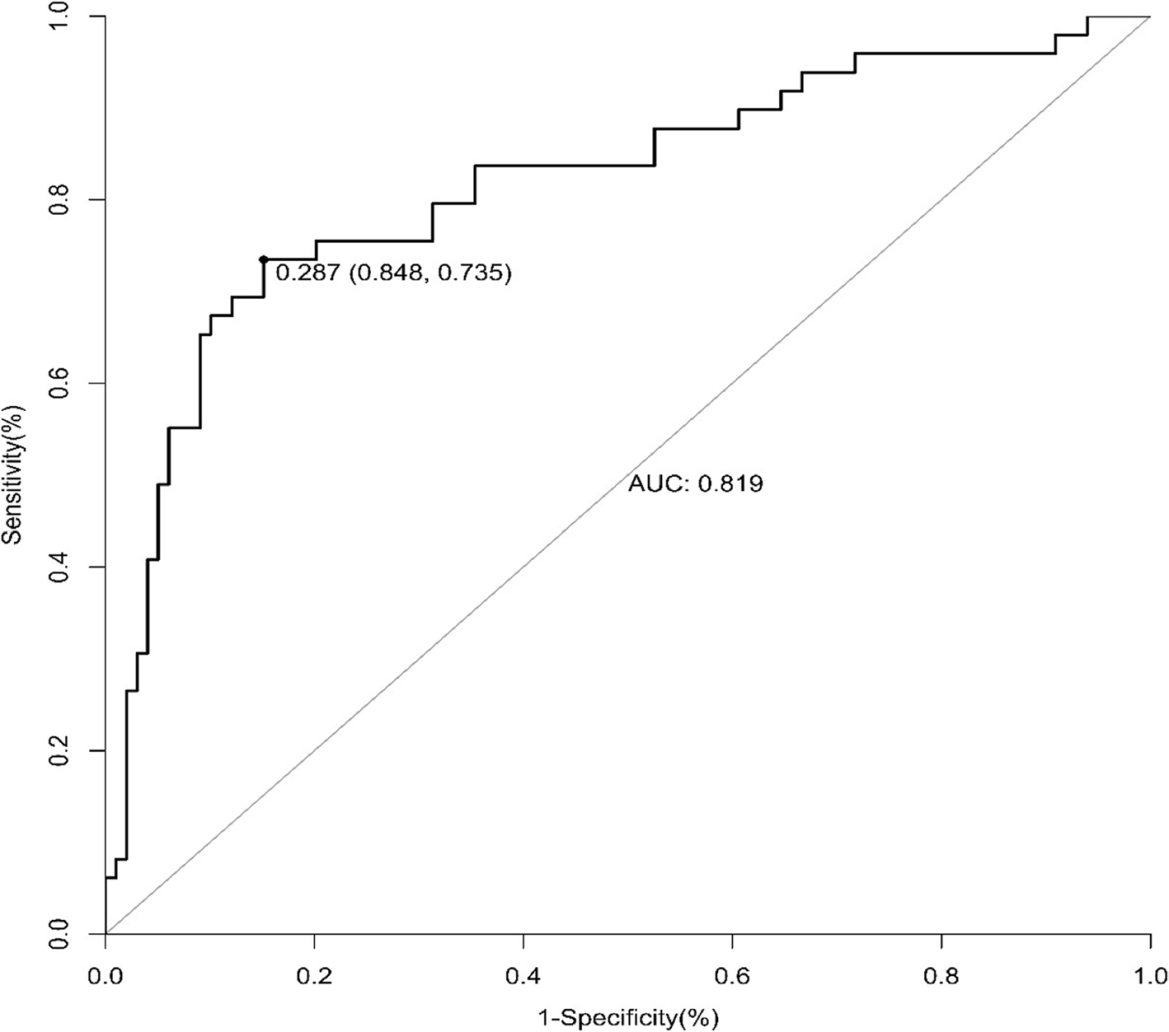
Receiver operating characteristic curve of peripartum blood transfusion in training set. AUC, area under the curve

**Fig. 3:**
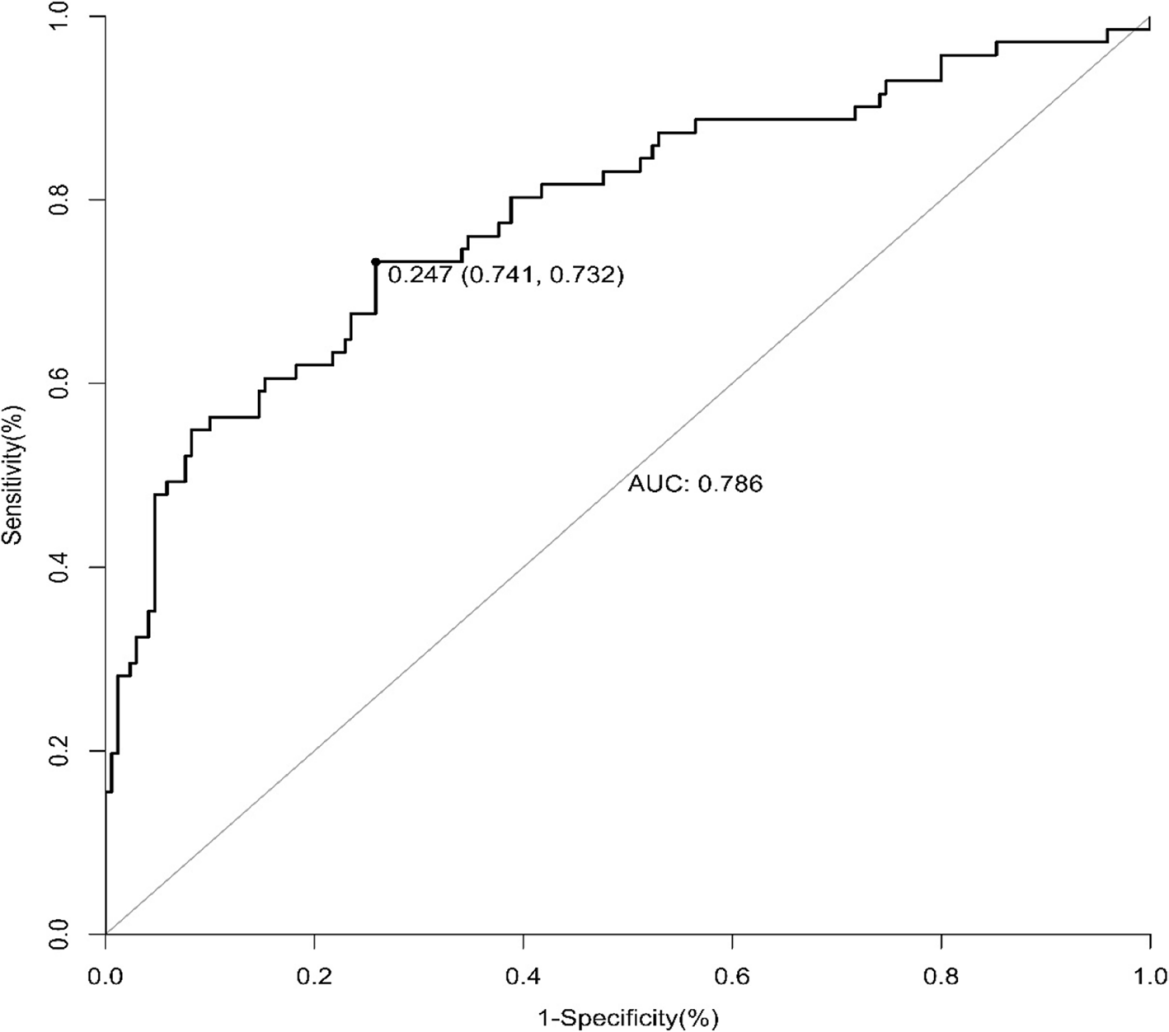
Receiver operating characteristic curve of peripartum blood transfusion in validation set. AUC, area under the curve

We used a ROC curve to evaluate the assessment of the model and to verify the model in the validation set. The results demonstrated that our model has acceptable discrimination (AUC: 0.819; sensitivity: 0.735; specificity: 0.848; critical value: 0.287) (Fig. 2). In the validation set, the internal verification results demonstrated that our validation model also has acceptable discrimination (AUC: 0.786; sensitivity: 0.732; specificity: 0.741; critical value: 0.247) (Fig. 3).

To verify the applicability and stability of the model, we used the Z-score test to compare the AUCs between the training set and the validation set. The results demonstrated that our model has good applicability and stability (*P* = 0.537; no statistically significant difference).

## Discussion

In this case–control study, we identified 5 risk factors for perinatal blood transfusion, including age, predelivery hemoglobin levels, predelivery fibrinogen levels, preeclampsia, and abnormal placentation. Preeclampsia and abnormal placentation are the two strongest risk factors in this study. When compared to a normal pregnancy, preeclampsia is characterized by the phenomena of systemic vascular resistance, lower cardiac output, and hypovolemia. Dehydrated pregnant women are vulnerable to hemodynamic instability, which is caused by PPH. An imbalance between angiogenic and antiangiogenic factors in the maternal blood is associated with gestational hypertension [18, 19]. In addition, a low platelet count and hypertension has been observed to aggravate blood loss and results in the requirement of transfusions. Preeclampsia is associated with placental ischemia, which consequently reduces the levels of placental growth factor, with increased coagulopathy resulting from the activation of the fibrinolytic system, platelet activation, and a decrease in platelet counts [20]. Prenatal anemia is one of the risk factors for perinatal blood transfusions and postpartum anemia [21, 22]. Abnormal coagulation is also an important cause of severe obstetric hemorrhage. Fibrinogen concentration is an important indicator for the evaluation of the coagulation function of pregnant women. This study confirmed that it can also be used as a predictive indicator for blood transfusions. Although there have been relevant studies on the risk factors for perinatal blood transfusions, there is a lack of clinical research for the development of risk assessment tools for perinatal blood transfusions.

This study involved more candidate predictors that may lead to perinatal blood transfusions than other studies, such as whether live in local, whether have permanent job, the number of previous deliveries, the number of abortions, the number of previous cesarean deliveries, anesthesia, emergency situations, fibroid levels, coagulation disorders, predelivery oxytocin use, and being hepatitis B virus carrier or abnormal liver function [23]. Besides, we established a risk prediction model of perinatal blood transfusions who underwent cesarean sections, using a nomogram. The AUC of the model was 0.819, which has good predictive performance and has been internally verified. When comparing the two AUCs, the model has good stability and applicability. The nomogram was able to integrate relevant risk factors and to quantify, visualize, and graph the logistic regression results. In addition, it was used to display continuous prediction probabilities and to personally predict the risks of clinical events. Furthermore, it is easy for clinical promotion and is expected to become a forecasting tool for perinatal blood transfusions.

When the nomogram predicts that the probability of a perinatal blood transfusion exceeds 50% (perhaps even 70%), it is suggested that sufficient preoperative preparations, adequate blood preparations, and a multidisciplinary cooperation is needed to initiate stabilization strategies, including circulatory support, as well as the maintenance of adequate tissue oxygenation, coagulation function, body temperature, and ionic equilibrium.

This study had some limitations. First, this was a retrospective analysis conducted at a single hospital with regional limitations. A multicentre and randomized controlled trial may help better evaluate the blood transfusion risks in patients. Second, although our model demonstrated good discrimination during internal validation, several important next steps are necessary, including the external validation of the model via the use of another cohort of patients. Third, the model established in this study only provides a risk assessment of whether a blood transfusion is required; it does not provide further predictions of required blood transfusion volumes. In addition, this model did not include those patients who required blood transfusions for treatment but were not transfused due to personal factors, although the number of these patients was extremely limited. More in-depth studies should therefore be conducted in the future.

## Conclusion

This study established a risk prediction model for perinatal blood transfusions in this region, using a nomogram. With the nomogram, the model can be further quantified and visualized, and clinical decision-making can subsequently be further simplified and promoted.

## Supplementary Information

**Additional file 1. **It is the training set to build risk prediction model.

**Additional file 2. **It is the validation set for the verification of the model.

## Data Availability

All data generated or analyzed during this study are included in this published article [and its supplementary information files].

## Abbreviations

PPH: Severe Postpartum Hemorrhage
PRBC: Packed Red Blood Cell
FFP: Fresh Frozen Plasma
HELLP: Hemolysis, Elevated Liver enzymes, and Low Platelets
GDM: Gestational Diabetes Mellitus
AUC: Area under the receiver operating characteristic curve
SPSS: Statistical Product and Service Solutions
ROC: Receiver Operating Characteristic
